# Has the increase in the regional nodes evaluated improved survival rates for patients with locoregional colon cancer?

**DOI:** 10.7150/jca.52352

**Published:** 2021-03-05

**Authors:** Zhongyi Zhou, Hong Zhu, Wenxue Liu, Fengbo Tan, Qian Pei, Lilan Zhao, Chenglong Li, Dan Wang, Yuan Zhou, Huan Peng, Haiping Pei, Yuqiang Li

**Affiliations:** 1 Department of General Surgery, Xiangya Hospital, Central South University, Changsha, China.; 2 Department of Radiotherapy, Xiangya Hospital, Central South University, Changsha, China.; 3 Department of Cardiology, Xiangya Hospital, Central South University, Changsha, China.; 4 Department of Rheumatology, Guangdong Provincial People's Hospital, Guangdong Academy of Medical Sciences, Guangzhou, China.; 5 Department of Thoracic surgery, Fujian Provincial Hospital, Fuzhou, China.; 6 Department of General Visceral and Thoracic Surgery, University Medical Center Hamburg-Eppendorf, Hamburg, Germany.

**Keywords:** colon cancer, regional nodes evaluated, surgery, surgical evolution, SEER database

## Abstract

**Background:** The guidelines for colon cancer surgery have been evolving over the past three decades. The advances in colectomy have focused mainly on the number of regional nodes evaluated (RNE).

**Methods:** Data in this retrospective analysis were extracted from the Surveillance, Epidemiology, and End Results (SEER) linked database.

**Results:** Rapid growth of RNE (the median rising from 10 (6-16) to 17 (13-23)) occurred from 2000 to 2009. The rate of colon cancer patients with positive lymph nodes following colectomy was greatly decreasing only in the group with RNE greater than 12 after 2000. Patients with T4 and/or N+ cannot obtain survival benefit from the increasing trend of RNE. The apparent survival benefit for T1-3N0 patients may result from augmented false negatives in patients from previous periods.

**Conclusions:** The golden period of surgical development in colon cancer, using RNE as an alternative indicator, occurred in the first decade of the 21st century. Although a more extensive lymph node evaluation is able to reduce the risk of underestimated staging, the increase of RNE does not provide survival benefits for locoregional colon cancer. A proper reduction in the scope of lymph node dissection may be reasonable in radical surgery for colon cancer.

## Introduction

The standards for colon cancer surgery have been evolving over the past three decades. The guidelines that were developed at the Sydney World Congress of Gastroenterology in 1990 stated that examination of at least 12 lymph nodes can be used as a benchmark to ensure proper resection and appropriate staging 1-3. Extended mesenteric excision for colon cancer was first proposed in 1997 4. In 2002, the American Joint Committee on Cancer (AJCC) established the identification of a minimum of 12 lymph nodes as the standard in colon cancer resection, which served as an impetus to increase the number of lymph nodes obtained and analyzed nationwide 5. Complete mesocolic excision (CME) was initially introduced in 2009 and then widely recognized by colorectal surgeons 6, 7.

Overall, the advances in colectomy mainly involved the number of regional nodes evaluated (RNE). Therefore, several studies have reported that the number of RNE has become a surrogate marker for the evaluation of the quality of surgery for patients with colon cancer 8, 9. What happened as a result of the trend in RNE during the progress of colectomy in the United States? When was the most rapid development period of colon cancer surgery? In addition, do patients with locoregional colon cancer obtain survival benefit from the changing RNE during the rapid development period? There is no final conclusion yet. Exploration of these issues may provide research directions related to colon cancer, or even all tumors, in the future.

Therefore, this study aimed to explore the trend of RNE from 1988 to 2016 in colectomy surgery for locoregional colon cancer and to compare the survival differences due to the evolution of RNE.

## Materials and Methods

### Patients

**Figure 1:**
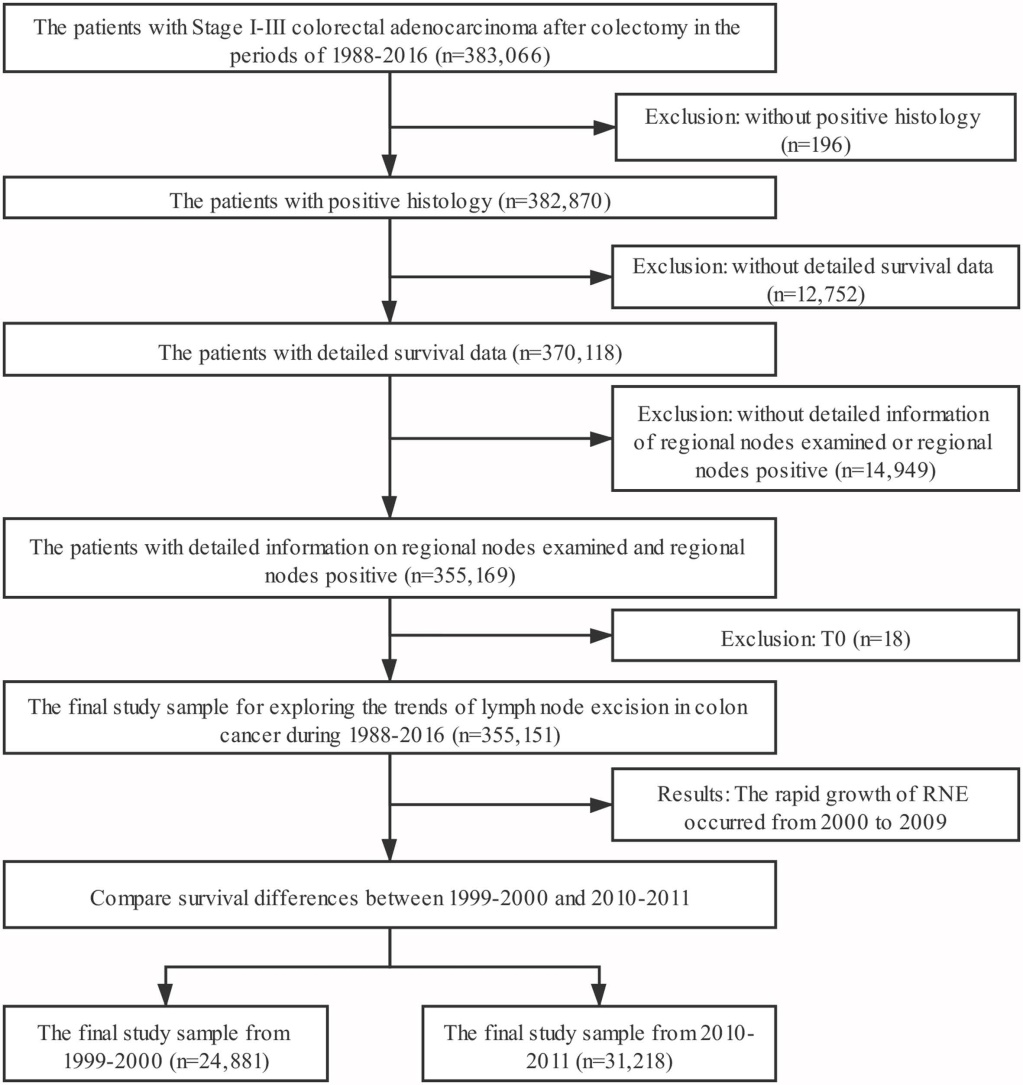
** The flow chart. Inclusion criteria:** the patients with Stage I-III colorectal adenocarcinoma after colectomy in the period 1988-2016 (n=383,066). **Exclusion criteria:** (1) without positive histology (n=196); (2) without detailed survival data (including survival months=0, diagnosed at autopsy or death certificate) (n=12,752); (3) without detailed information of regional nodes examined (including RNE=0) or regional nodes positive (n=14,949); (4) T0 (n=18).

Data in this retrospective analysis were extracted from the Surveillance, Epidemiology, and End Results (SEER) linked database. The SEER Program of the National Cancer Institute is an authoritative source of information on cancer incidence and survival in the United States that is updated annually. SEER currently collects and publishes cancer incidence and survival data from population-based cancer registries covering approximately 34.6% of the U.S. population. Our target population were the patients with Stage I-III colorectal adenocarcinoma after colectomy in the period 1988-2016 (n=383,066). Exclusion criteria: (1) without positive histology (n=196); (2) without detailed survival data (including survival months=0, diagnosed at autopsy or death certificate) (n=12,752); (3) without detailed information of regional nodes examined (including RNE=0) or regional nodes positive (n=14,949); (4) T0 (n=18); leading to a sample of 355,151 patients. The target population for survival analysis was limited to patients in the periods of 1999-2000 and 2010-2011. The third edition AJCC staging was adopted in colorectal cancer in 1999-2000; however, the sixth edition of the AJCC staging was applied to for the patients of 2009-2010. Therefore, we re-staged the N stage according to the number of positive lymph nodes. We defined N1 as 1-3 lymph nodes positive and N2 as more than 4 lymph nodes positive. The final study sample contained 56,099 patients (Fig. 1).

### Methods

Intergroup comparisons were performed with the Pearson's chi-square test or Mann-Whitney U-test, depending on the nature of the data. Log-rank test was used to compare overall survival (OS) between different groups. A hazard ratio (HR) and a 95% confidence interval (CI) were evaluated by a single factor and a multivariate Cox proportional hazards regression model. The variables with significant differences in univariate analysis were included in the Cox regression model for multivariate analysis. In order to eliminate the influence of other variables, we conducted a propensity score matching (PSM). The nearest neighbor matching with a caliper width of 0.0001 was employed. Statistical analyses were performed with IBM SPSS statistics trial ver. 25.0 (IBM, Armonk, NY, USA). All reported p-values lower than 0.05 were considered significant.

## Results

### The trends of RNE during 1988 to 2016

**Figure 2:**
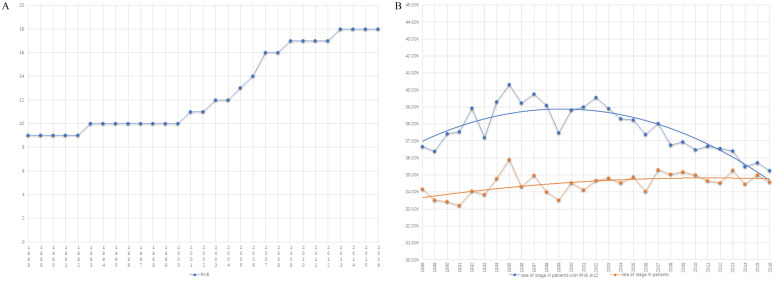
The trend graph. **A)** The trend of the number of RNE from 1988-2016. **B)** The trend of the rate of colon cancer patients with positive lymph nodes (orange line: the proportion of the entire patients with stage III colon cancer; blue line: the rate of colon cancer patients with positive lymph nodes in those with RNE≥ 12).

Table 1 summarizes the number of cases from 1988 to 2016. There are two platform periods and one growth period displaying the trend of RNE (Fig. 2A). The median number of RNE increased from 9 (5-15) to 10 (6-16) in the first platform period of 1988-2000. The median of RNE also increased by only one (from 17 (13-23) to 18 (14-24)) in the second platform period (from 2009 to 2016). The rapid growth of RNE (the increase in the median from 10 (6-16) to 17 (13-23)) occurred in the period 2000 to 2009, which suggests that the golden period for the development of surgery was in the first decade of the 21st century (Fig. 2A).

Increasing RNE did not cause a significant rise in the proportion of patients with stage III colon cancer. In fact, the rate of colon cancer patients with positive lymph nodes had greatly decreased after 2000 when only comparing patients with RNE greater than 12 (Fig. 2B). The obvious changes attracted us to compare survival differences between 1999-2000 with those in 2010-2011, as described in the following section.

### Cox regression analysis

In this section, which includes cases with locoregional colon cancer from 1999-2000 and 2010-2011, the focus was on comparing survival differences due to the rising trend of RNE. Table 2 displays the characteristics of the patients in this section. There was significant difference in RNE between the two periods. The Cox regression analysis did not include RNE because it could have interfered with results caused by the changing trend. Meanwhile, the year of diagnosis was used as a variable to replace the trend of RNE. Moreover, the significant variables in the univariate analysis were brought into the multivariate analysis.

**Figure 3:**
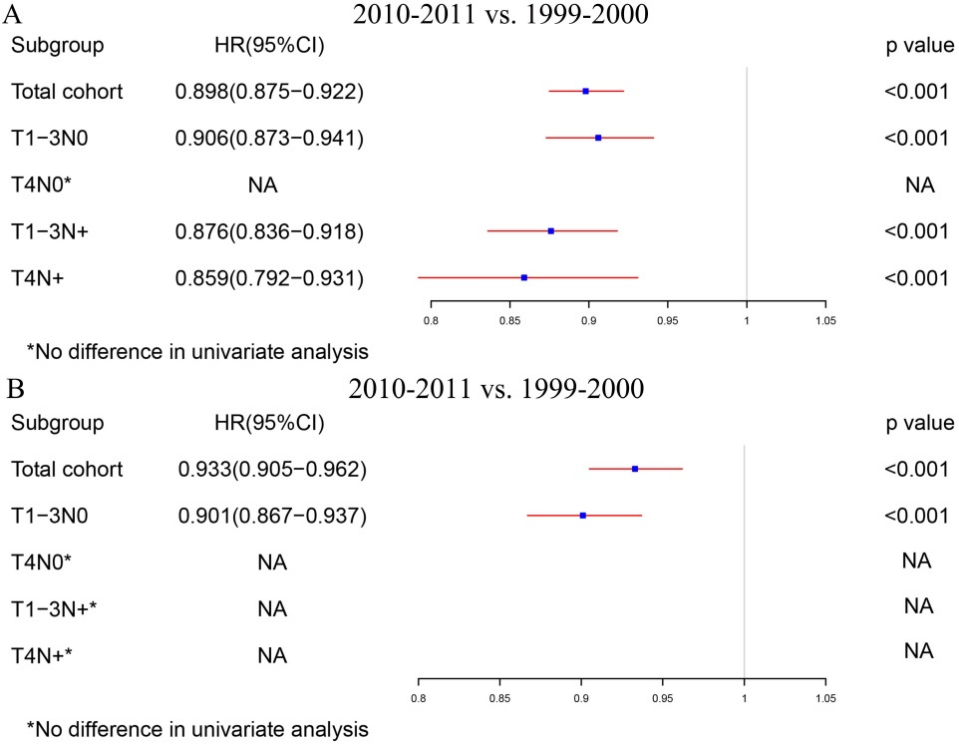
The survival differences in multivariate COX regression analysis (2010-2011 vs. 1999-2000). **A.** The survival differences of the year of diagnosis in all locoregional colon cancer patients. **B.** The survival differences of the year of diagnosis in locoregional colon cancer patients without chemotherapy.

The total cohort and the four subgroups, including T1-3N0, T4N0, T1-3N+ and T4N+, were analyzed separately for all patients. Table S1 shows the detailed results in the univariable and multivariable Cox regression model of all locoregional colon cancer patients in the two periods. Fig. 3A summarizes the survival differences by the year of diagnosis. Locoregional colon cancer patients from 2010-2011, as an entirety, had better survival than those from 1999-2000. Meanwhile, the subgroup analysis displayed that colon cancer patients with T1-3N0 and T_any_N+ from 2010-2011 had superior survival to those from 1999-2000. However, there was no significant difference between the two periods in the colon cancer patients with T4N0.

In order to rule out the impact of the advances in chemotherapy, we further analyzed those patients who did not receive chemotherapy. Table S2 indicates the exhaustive results of the univariable and multivariable Cox regression model for patients without chemotherapy. Similar to the total group results, locoregional colon cancer patients without chemotherapy from 2010-2011 had better survival than those from 1999-2000 in the analysis of this cohort. However, only T1-3N0 patients without chemotherapy can obtain survival benefit from the increased RNE in the subgroup analysis (Fig. 3B).

### Propensity score matching

**Figure 4:**
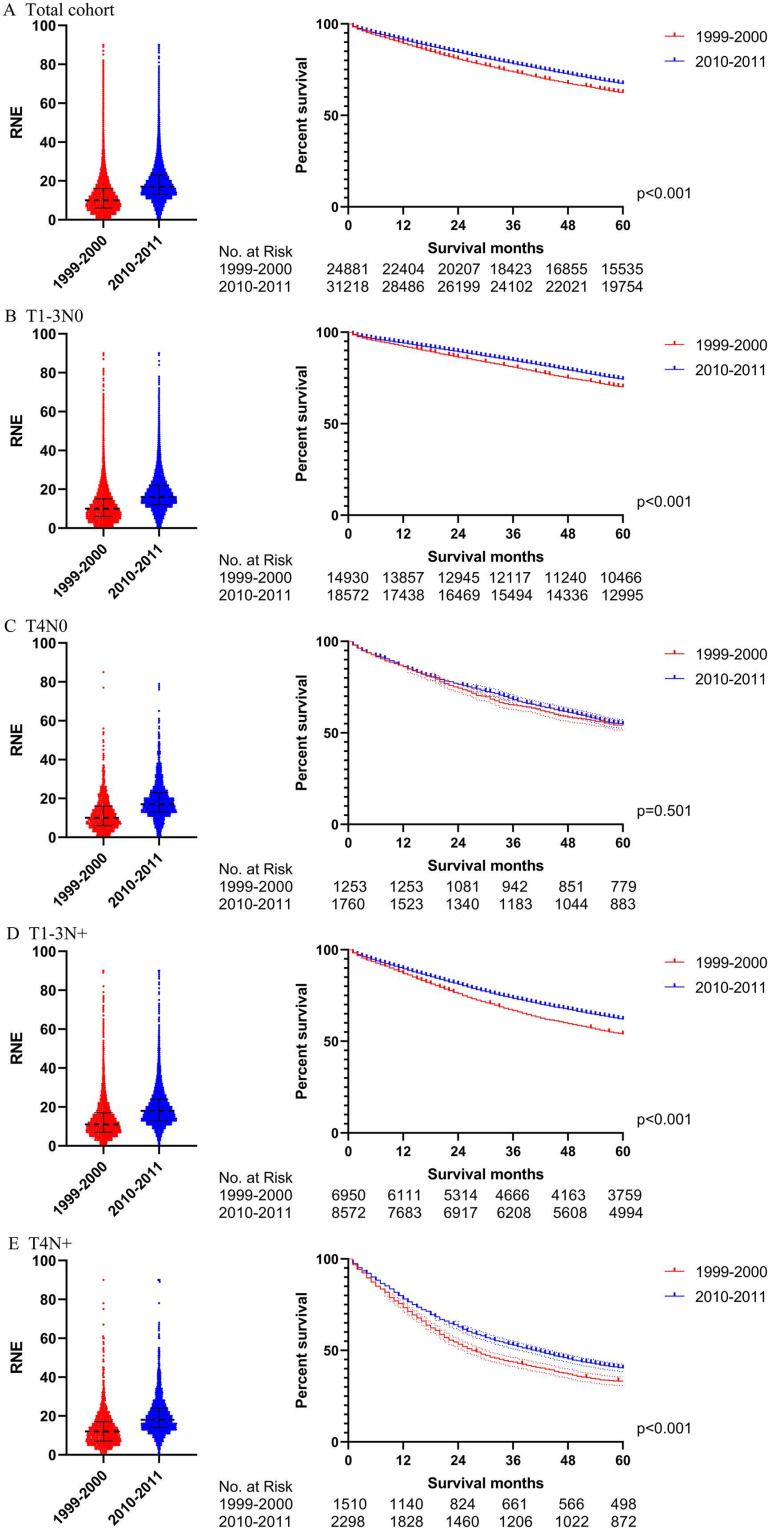
The survival analysis before PSM in all locoregional colon cancer patients (left: RNE of each patient, all p<0.001; right: survival curve). **A.** The total cohort. **B.** T1-3N0. **C.** T4N0. **D.** T1-3N+.** E.** T4N+.

**Figure 5:**
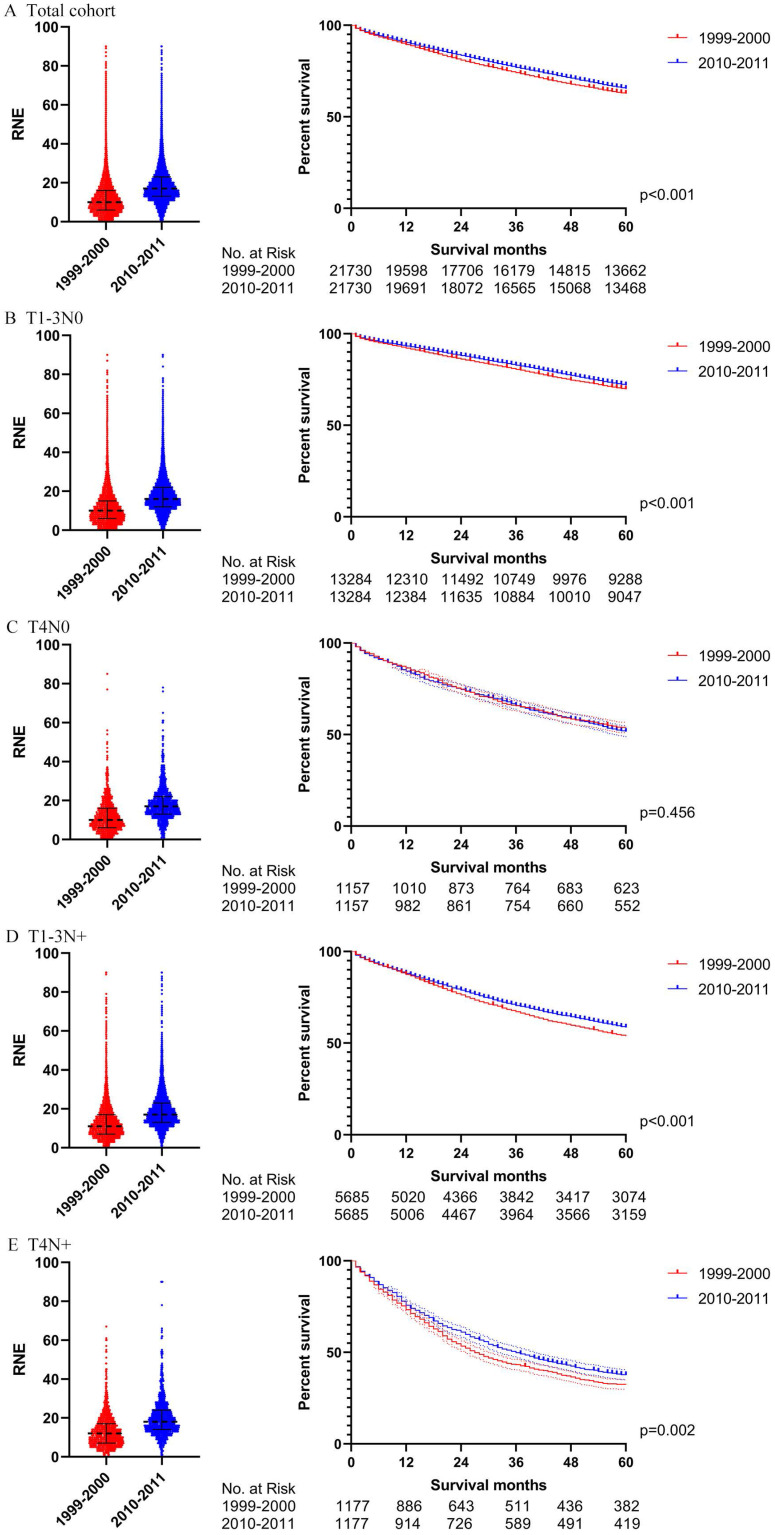
The survival analysis after PSM in all locoregional colon cancer patients (left: RNE of each patient, all p<0.001; right: survival curve). **A.** The total cohort. **B.** T1-3N0. **C.** T4N0. **D.** T1-3N+.** E.** T4N+.

In order to verify the results of the Cox regression analysis, we conducted propensity score matching (PSM) to eliminate the influence of other variables. Table S3 illustrates the characteristics of all the patients with locoregional colon cancer following PSM. The number of RNE, which can reflect the quality of surgery, did not match between the two periods. Differences in survival before PSM were similar to those after PSM in both the total cohort and the subgroups (Figs. 4 and 5). The total cohort received survival benefit from the increased RNE. Colon cancer patients with T1-3N0 and T_any_N+ from 1999-2000 suffered a worse survival compared with those from 2010-2011. However, T4N0 colon cancer patients did not obtain survival benefit from the increased RNE either before (p=0.501) or after PSM (p=0.456).

**Figure 6:**
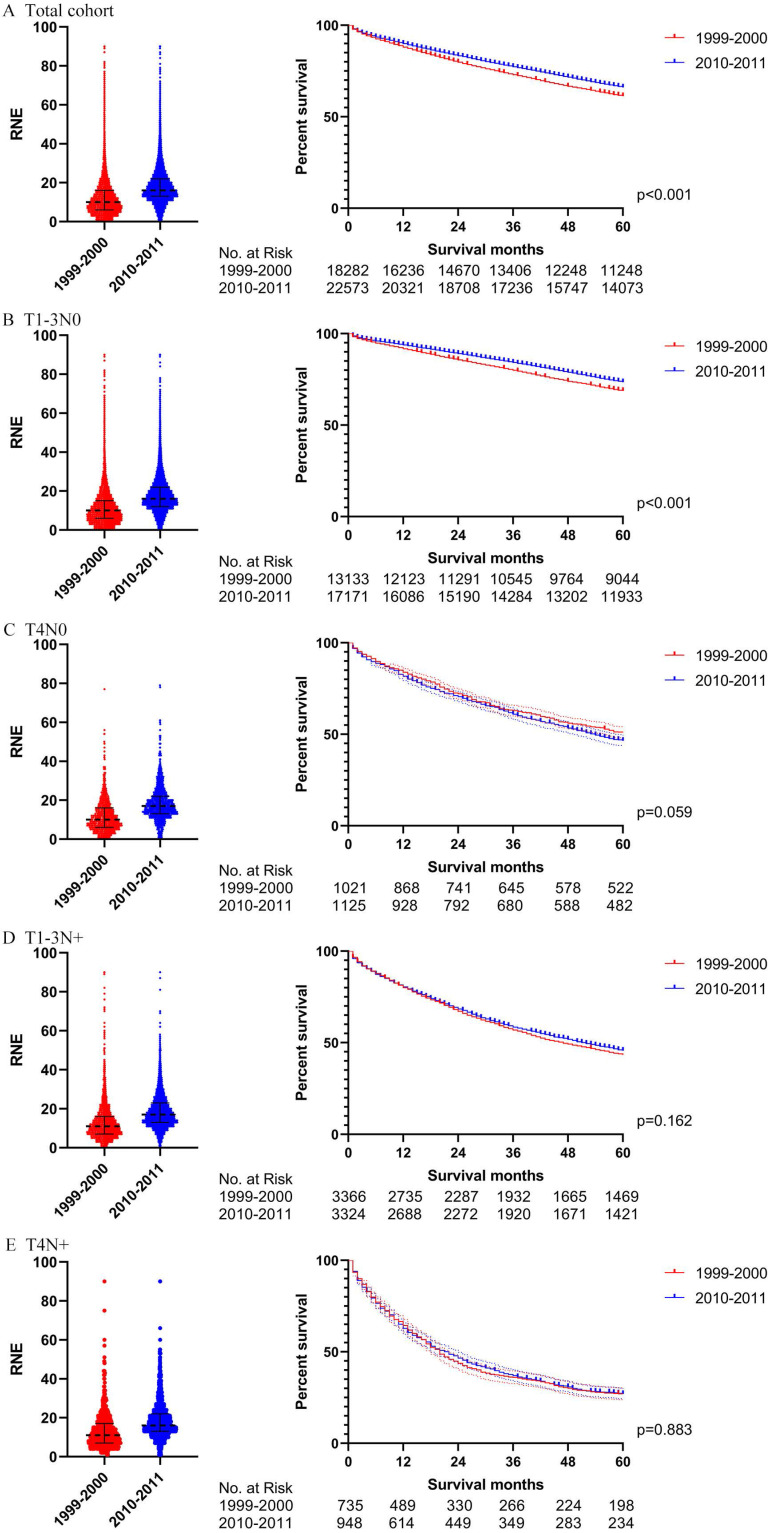
The survival analysis before PSM in locoregional colon cancer patients without chemotherapy (left: RNE of each patient, all p<0.001; right: survival curve). **A.** The total cohort. **B.** T1-3N0. **C.** T4N0. **D.** T1-3N+.** E.** T4N+.

**Figure 7:**
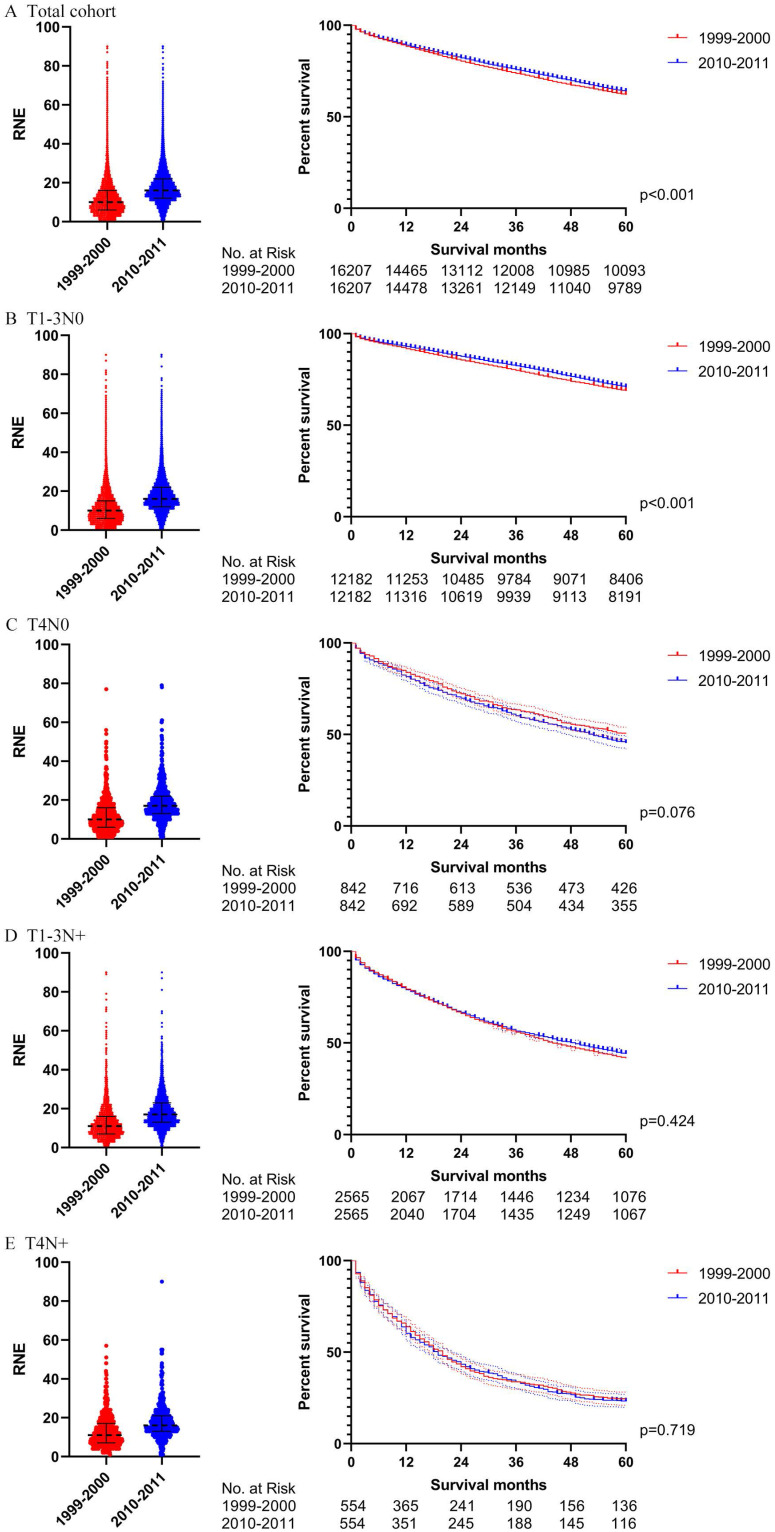
The survival analysis before PSM in locoregional colon cancer patients without chemotherapy (left: RNE of each patient, all p<0.001; right: survival curve). **A.** The total cohort. **B.** T1-3N0. **C.** T4N0. **D.** T1-3N+.** E.** T4N+.

Then we further analyzed the locoregional colon cancer patients without chemotherapy. Table S4 describes the characteristics of the patients without chemotherapy after PSM. The results after PSM were consistent with those before PSM (Figs. 6 and 7). Locoregional colon cancer patients without chemotherapy from 2010-2011 showed a superior survival rate in the total cohort analysis. There were significant survival differences in colon cancer patients with T1-3N0 who did not receive chemotherapy between the two periods (p<0.001 both before and after PSM). However, patients with T4 and/or N+ who missed chemotherapy did not obtain survival benefit from the increasing trend of RNE either before or after PSM.

### Further analysis of T1-3N0 patients with RNE greater than 12

**Figure 8:**
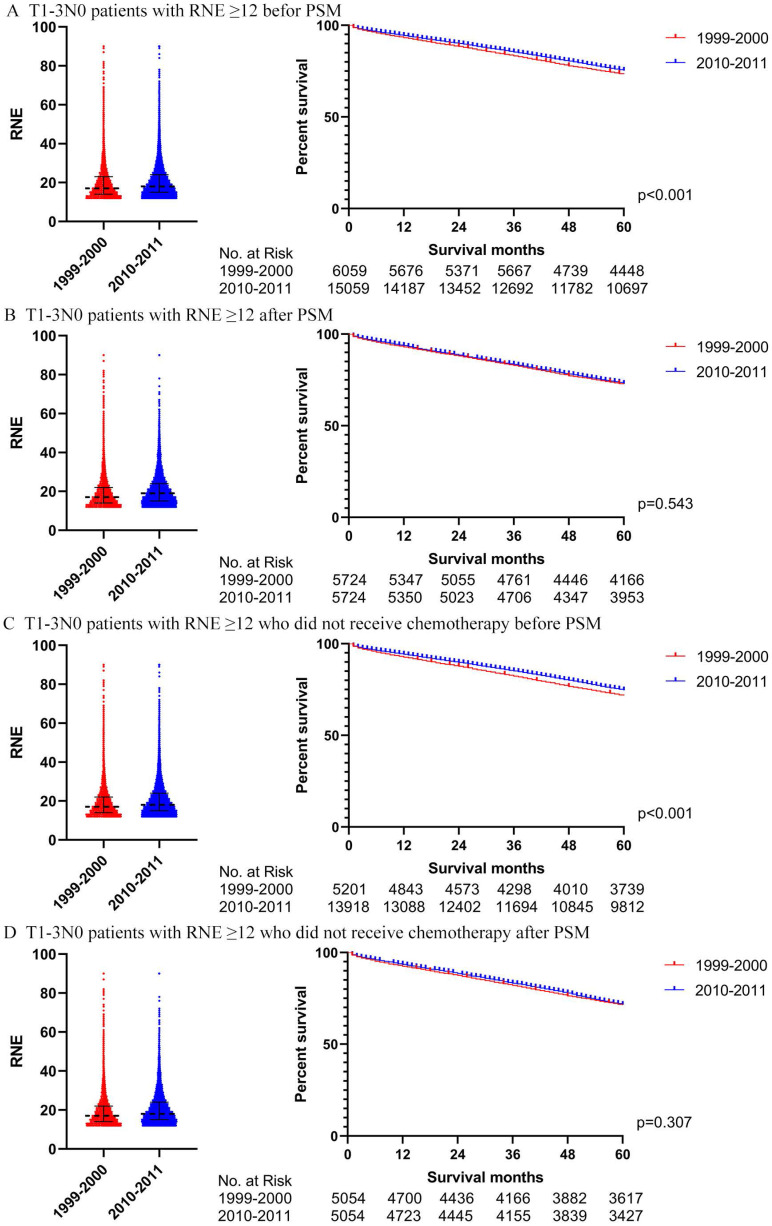
The survival analysis in T1-3N0 colon cancer patients with RNE≥ 12 (left: RNE of each patient, all p<0.001; right: survival curve). **A.** T1-3N0 colon cancer patients with RNE≥ 12 before PSM. **B.** T1-3N0 colon cancer patients with RNE≥ 12 after PSM. **C.** T1-3N0 colon cancer patients with RNE≥ 12 who did not receive chemotherapy before PSM. **D.** T1-3N0 colon cancer patients with RNE≥ 12 who did not receive chemotherapy after PSM.

We also utilized Cox regression analysis and propensity score matching to explore whether T1-3N0 patients with RNE greater than 12 from 2010-2011 could achieve a winning survival compared to those from 1999-2000. Table 3 displays the characteristics of the T1-3N0 patients with RNE greater than 12 from 1999-2000 and from 2010-2011. The RNE of patients from 2010-2011 was slightly larger than that from 1999-2000 (18 (15-24) vs. 17(14-23), p<0.001). However, the multivariate Cox regression analysis, for both of the entire patient group (p=0.138) and patients without chemotherapy (p=0.091), did not demonstrate significant differences between the two periods (Table S5). Table S6 illustrates the characteristics of the T1-3N0 patients with RNE≥12 after PSM. Survival differences between 1999-2000 and 2010-2011 tapered from significant differences before PSM (both the entire group of patients and patients without chemotherapy: p <0.001) to insignificant after PSM (the entire group of patients: p=0.543; the patients without chemotherapy: p=0.307) (Fig. 8).

## Discussion

This population-based study confirmed one growth period and two platform periods regarding the trend in the number of RNE during colectomy for locoregional colon cancer in the past decades. The rapidly growing RNE revealed that the period from 2000 to 2009 was the golden phase of the development of colon cancer surgery. Previous research has reported that increasing RNE was not able to improve the staging of colon cancer 5. However, this study believes that improvements in colorectal cancer screening and early detection can lead to patients being diagnosed at an earlier stage than in previous years because the proportion of patients with colon cancer with positive lymph nodes showed a downward trend in patients with RNE greater than 12, which allows assessing lymph node staging more accurately 10. Moreover, although several previous studies reported that RNE showed an increasing trend in colectomy, they failed to discuss the impact of this tendency on survival for colon cancer 8, 11-14. Collectively, these changes attracted us to explore the survival differences before and after the golden period of colon cancer surgery. Therefore, this study focused on comparing survival differences between patients from the period 1999-2000, as a baseline, to 2010-2011, which included patients with the most recent 5-year follow-up.

An increasing number of studies, making horizontal comparisons, have reported better survival for patients with more lymph nodes evaluated among those surgically treated for colon cancer 15-17. Our study, longitudinally comparing survival differences associated with greater RNE, seems to support this viewpoint that patients with locoregional colon cancer from 2010-2011 achieved superior survival to those from 1999-2000 in the analysis of the total cohort. However, this conclusion is due to the fact that T1-3N0, representing the vast majority of locoregional colon cancer, masked the situation that locally advanced colon cancer (T4 and/or N +) cannot obtain benefit from increasing RNE. Research without subgroup analysis 8, 18 may come to such erroneous conclusions. Moreover, studies that analyze patients with stage II colon cancer as a whole 19, may also miss the true condition of T4N0 patients. Meanwhile, patients with stage III colon cancer were able to gain survival benefit from the advanced chemotherapy regimen, evolving from 5-FU/leucovorin in the 1999-2000 20 to FOLFOX (oxaliplatin/5-FU/leucovorin) in 2010-2011 9, demonstrated in this study by the significant survival differences that appeared in the overall analysis of stage III but not in those patients who did not receive chemotherapy. An analysis of the SEER database also supported that advancements in chemotherapy were the main contributor to the upswing in the survival of locally advanced colon cancer 9. Unfortunately, patients with T4N0 did not acquire survival benefits from the improved chemotherapy and surgery. Therefore, patients with T4N0, who actually suffered worse survival compared to those with IIIA stage colon cancer 21, should gain more attention in future clinical practice.

In addition, the reason that T1-3N0 patients received survival benefit from the increasing RNE may also be due to higher increased false negatives in patients from 1999 to 2000. A more extensive lymph node evaluation is able to reduce the risk of underestimated staging, in which inadequate assessment may incorrectly identify patients with node-positive disease as node-negative, resulting in failure to identify appropriate treatment. In 1999-2000, the proportion of patients with positive lymph nodes among those with RNE greater than 12 was much larger than that among the overall group. In fact, such a difference gradually decreased after 2000. The proportion of patients with RNE fewer than 12 reached 56.37% in 1999-2000, which may have caused the proportion of patients with lymph node-positive to be greatly underestimated. Furthermore, the additional analysis involving the patients with RNE greater than 12 showed that real T1-3N0 patients from 2010-2011 did not have superior survival compared to those from 1999-2000. Therefore, it is reasonable that the real T1-3N0 patients will not benefit from the advancements of surgery.

Increasing RNE played an important role in accurate assessment of the N stage of colon cancer. However, too much RNE is not able to provide better long-term survival, or even reduce the short-term survival 9. Unfortunately, there is always a constant emphasis on extensive lymph node evaluation during the evolution of colon cancer surgery. However, several scholars have begun to question whether expanding lymph node dissection, including the concept of CME, is better than traditional D2 resection 22. Another controversy about aggressive D3 resection versus imperturbable D2 resection has appeared in the radical operation of gastric cancer. The measurement of surgical risk and survival benefit promoted that D2 resection should be the standard procedure for gastric cancer. The experiences from gastric cancer suggest that properly reducing the scope of lymph node dissection may be reasonable for radical surgery in colon cancer.

Numerous surgeons would rather kill more negative nodes instead of missing even one positive node during the radical surgery for colon cancer, which is one of the reasons why CME surgery has been recognized widely. However, the increasing RNE cannot provide survival benefit to colon patients including those with positive nodes. Can the serious consequences of missing metastatic lymph nodes be compensated for by chemotherapy? The fact that neoadjuvant chemotherapy may lead to the downstaging of a part of colon cancer supports that chemotherapy is capable of killing cancer cells in regional lymph nodes. Meanwhile, combination chemotherapy can improve the pathological complete response rate of colorectal cancer 23, 24. Therefore, advanced chemotherapy, but not advancements in colectomy, can better compensate for the consequences of missing positive lymph nodes. In fact, colon cancer with positive lymph nodes should be regarded as a systemic disease that cannot be cured by surgery alone. Advances in chemotherapy regimens are able to provide a better rationale for properly reducing the scope of lymph node dissection.

Although population-based studies such as this offer increased statistical power and generalizability of results, this study was limited by lack of data on comorbidities and clinical presentation of the patient (i.e. cancer found on screening or due to symptoms). There were several limitations in the SEER database such as unrecorded variables, incomplete data regarding detailed adjuvant therapy, variations in the way of recording data, and migration of patients into and out of SEER registry areas. Furthermore, various studies from different countries have also displayed an increasing number of RNE in colectomy during the first decade of the 21st century 8, 13, 14, which supports the results of the trends of RNE using U.S. data. However, the survival results of other populations need to be further verified by more clinicians from different regions.

## Conclusions

The golden period of surgical development in colon cancer, using RNE as an alternative indicator, occurred in the first decade of the 21st century. Although a more extensive lymph node evaluation is able to reduce the risk of underestimated staging, increasing RNE does not provide survival benefits for locoregional colon cancer. A cautious reduction in the scope of lymph node dissection may be reasonable for radical surgery of colon cancer.

## Synopsis

This population-based study confirmed one growth period and two platform periods regarding the trend of the number of RNE during colectomy for locoregional colon cancer in the past decades.

Although a more extensive lymph node evaluation is able to reduce the risk of underestimated staging, the increasing RNE cannot provide survival benefits for locoregional colon cancer.

## Supplementary Material

Supplementary tables.

## Figures and Tables

**Table 1:** The trends of the number of RNE from 1988 to 2016

Year	No. of cases	No. of stage III cases	Rate of stage III	No. of cases with RNE ≥12	Rate of cases with RNE ≥12	No. of stage III cases with RNE ≥ 12	Rate of stage III in cases with RNE ≥ 12	RNE
1988	4809	1643	34.17%	1803	37.49%	661	36.66%	9 (5-15)
1989	4820	1616	33.53%	1797	37.28%	654	36.39%	9 (5-15)
1990	5197	1737	33.42%	2031	39.08%	760	37.42%	9 (5-15)
1991	5410	1796	33.20%	2182	40.33%	819	37.53%	9 (5-15)
1992	7168	2441	34.05%	2728	38.06%	1062	38.93%	9 (5-15)
1993	7078	2396	33.85%	2903	41.01%	1080	37.20%	10 (6-15)
1994	7226	2514	34.79%	2894	40.05%	1137	39.29%	10 (6-15)
1995	7253	2604	35.90%	2875	39.64%	1159	40.31%	10 (6-15)
1996	7422	2546	34.30%	3107	41.86%	1219	39.23%	10 (6-16)
1997	7803	2730	34.99%	3371	43.20%	1340	39.75%	10 (6-16)
1998	8224	2796	34.00%	3574	43.46%	1397	39.09%	10 (6-16)
1999	8160	2735	33.52%	3609	44.23%	1352	37.46%	10 (6-16)
2000	16721	5776	34.54%	7247	43.34%	2812	38.80%	10 (6-16)
2001	17275	5895	34.12%	7956	46.05%	3102	38.99%	11 (7-16)
2002	17314	6004	34.68%	8421	48.64%	3330	39.54%	11 (7-17)
2003	17180	5981	34.81%	8711	50.70%	3390	38.92%	12 (7-18)
2004	16833	5816	34.55%	9111	54.13%	3489	38.29%	12 (8-18)
2005	16242	5665	34.88%	9549	58.79%	3650	38.22%	13 (8-19)
2006	16673	5672	34.02%	10738	64.40%	4014	37.38%	14 (9-20)
2007	16783	5922	35.29%	12264	73.07%	4665	38.04%	16 (11-22)
2008	16905	5925	35.05%	13083	77.39%	4810	36.77%	16 (12-22)
2009	16411	5773	35.18%	13341	81.29%	4929	36.95%	17 (13-23)
2010	15749	5514	35.01%	12989	82.48%	4737	36.47%	17 (13-23)
2011	15469	5359	34.64%	12982	83.92%	4762	36.68%	17 (13-23)
2012	15496	5351	34.53%	13296	85.80%	4861	36.56%	17 (13-23)
2013	15261	5385	35.29%	13368	87.60%	4868	36.42%	18 (14-24)
2014	15429	5319	34.47%	13754	89.14%	4880	35.48%	18 (14-24)
2015	15053	5268	35.00%	13518	89.80%	4828	35.72%	18 (14-24)
2016	13787	4767	34.58%	12526	90.85%	4416	35.25%	18 (14-24)

**Table 2:** The characteristics of colon cancer patients in 1999-2000 and 2010-2011

Characteristics	1999-2000 (n=24881)		2010-2011 (n=31218)		p
	N	%	N	%	
**Gender**					0.008
Female	13066	52.51%	16042	51.39%	
Male	11816	47.49%	15176	48.61%	
**Age (years)**					<0.001
≤50	1881	7.56%	3156	10.11%	
51-65	5626	22.61%	8865	28.40%	
>65	17374	69.83%	19197	61.49%	
**Marital status**					0.017
Married	13359	53.69%	16446	52.68%	
Unmarried/NOS	11522	46.31%	14772	47.32%	
**Race**					<0.001
White	20768	83.47%	24896	79.75%	
Black	2373	9.54%	3652	11.70%	
Other/NOS	1740	6.99%	2670	8.55%	
**Tumor location**					<0.001
Right colon	14981	60.21%	19385	62.10%	
Left colon	9522	38.27%	11332	36.30%	
NOS	378	1.52%	501	1.60%	
**Pathological grade**					<0.001
I/II	19055	76.58%	24521	78.55%	
III/IV	4951	19.90%	5807	18.60%	
Unknown	875	3.52%	890	2.85%	
**Histological type**					<0.001
Adenocarcinomas	21279	85.52%	27999	89.69%	
MCC/SRCC	3602	14.48%	3219	10.31%	
**T stage**					<0.001
T1	2952	11.86%	4809	15.40%	
T2	3964	15.93%	5258	16.84%	
T3	14964	60.14%	17077	54.70%	
T4	2949	11.85%	4058	13.00%	
Tx	52	0.21%	16	0.05%	
**N stage**					0.001
N0	16370	65.79%	20333	65.13%	
N1	5788	23.26%	7074	22.66%	
N2	2723	10.94%	3811	12.21%	
**Chemotherapy**					0.002
Yes	6599	26.52%	8645	27.69%	
No	18282	73.48%	22573	72.31%	
RNE	10 (6-16)		17 (13-23)		<0.001

MCC: mucinous cell carcinoma; SRCC: signet ring cell carcinoma; NOS: not otherwise specified.

**Table 3:** The Characteristics of the T1-3N0 patients with RNE greater than 12 in 1999-2000 and 2010-2011

Characteristics	1999-2000 (n=6059)		2010-2011 (n=15059)		*p*
	N	%	N	%	
**Gender**					0.027
Female	3253	53.69%	7832	52.01%	
Male	2806	46.31%	7227	47.99%	
**Age (years)**					<0.001
≤50	527	8.70%	1311	8.71%	
51-65	1346	22.21%	4171	27.70%	
>65	4186	69.09%	9577	63.60%	
**Marital status**					0.780
Married	3218	53.11%	8030	53.32%	
Unmarried/NOS	2841	46.89%	7029	46.68%	
**Race**					<0.001
White	5126	84.60%	12279	81.54%	
Black	550	9.08%	1666	11.06%	
Other/NOS	383	6.32%	1114	7.40%	
**Tumor location**					0.001
Right colon	4233	69.86%	10084	66.96%	
Left colon	1725	28.47%	4785	31.78%	
NOS	101	1.67%	190	1.26%	
**Pathological grade**					<0.001
I/II	4806	79.32%	12632	83.88%	
III/IV	1064	17.56%	2005	13.31%	
Unknown	189	3.12%	422	2.80%	
**Histological type**					<0.001
Adenocarcinomas	5186	85.59%	13680	90.84%	
MCC/SRCC	873	14.41%	1379	9.16%	
**T stage**					<0.001
T1	713	11.77%	2935	19.49%	
T2	1258	20.76%	3514	23.33%	
T3	4088	67.47%	8610	57.18%	
**Chemotherapy**					<0.001
Yes	858	14.16%	1141	7.58%	
No	5201	85.84%	13918	92.42%	
RNE	17 (14-23)		18 (15-24)		<0.001

MCC: mucinous cell carcinoma; SRCC: signet ring cell carcinoma; NOS: not otherwise specified.
