# Supplementary material for: Has the increase in the regional nodes evaluated improved survival rates for patients with locoregional colon cancer?
Source: J Cancer. 2021 Mar 5;12(9):2513–25. doi: 10.7150/jca.52352 (PMC8040710; doi:10.7150/jca.52352)
Supplement: Supplementary file 1 — Supplementary tables. [file jcav12p2513s1.pdf]

Table S1 Univariable and multivariable Cox regression model in all patients

**Total cohort**								
Characteristics	Univariable analysis				Multivariable analysis			
	HR	95% CI lower	95% CI upper	p-value	HR	95% CI lower	95% CI upper	p-value
**Year of diagnosis**				**<0.001**				**<0.001**
**1999-2000**		**reference**				**reference**		
**2010-2011**	**0.841**	**0.819**	**0.863**	**<0.001**	**0.898**	**0.875**	**0.922**	**<0.001**
Gender				0.036				<0.001
Female		reference				reference		
Male	1.025	1.002	1.048	0.036	1.255	1.225	1.286	<0.001
Age(years)				<0.001				<0.001
≤50		reference				reference		
51-65	1.474	1.382	1.571	<0.001	1.553	1.456	1.656	<0.001
>65	3.872	3.650	4.106	<0.001	3.864	3.640	4.101	<0.001
Marital status				<0.001				<0.001
Married		reference				reference		
Unmarried/NOS	1.459	1.426	1.493	<0.001	1.381	1.348	1.415	<0.001
Race				<0.001				<0.001
White		reference				reference		
Black	0.984	0.948	1.022	0.402	1.123	1.082	1.167	<0.001
Other/NOS	0.762	0.727	0.798	<0.001	0.814	0.776	0.853	<0.001
Tumor location				<0.001				0.005
Right colon		reference				reference		
Left colon	0.806	0.787	0.826	<0.001	0.972	0.948	0.996	0.023
NOS	1.154	1.058	1.259	0.001	1.096	1.004	1.196	0.039
Pathological grade				<0.001				<0.001
I/II		reference				reference		
III/IV	1.345	1.309	1.383	<0.001	1.139	1.107	1.172	<0.001
Unknown	0.866	0.809	0.927	<0.001	1.008	0.940	1.081	0.821
Histological type				<0.001				0.022
Adenocarcinomas		reference				reference		
MCC/SRCC	1.190	1.152	1.230	<0.001	1.040	1.006	1.075	0.022
T stage				<0.001				<0.001
T1		reference				reference		
T2	1.299	1.239	1.361	<0.001	1.178	1.123	1.236	<0.001
T3	1.700	1.635	1.769	<0.001	1.486	1.426	1.548	<0.001
T4	2.630	2.511	2.755	<0.001	2.317	2.206	2.434	<0.001
Tx	1.722	1.283	2.311	<0.001	1.148	0.855	1.543	0.359
N stage				<0.001				<0.001

N0		reference				reference		
N1	1.253	1.219	1.288	<0.001	1.449	1.406	1.493	<0.001
N2	1.968	1.904	2.034	<0.001	2.346	2.261	2.435	<0.001
Chemotherapy				<0.001				<0.001
Yes		reference				reference		
No	1.272	1.239	1.306	<0.001	1.546	1.500	1.594	<0.001
**T1-3N0**								
**Year of diagnosis**				**<0.001**				**<0.001**
**1999-2000**		**reference**				**reference**		
**2010-2011**	**0.835**	**0.804**	**0.867**	**<0.001**	**0.906**	**0.873**	**0.941**	**<0.001**
Gender				<0.001				<0.001
Female		reference				reference		
Male	1.062	1.030	1.095	<0.001	1.303	1.261	1.346	<0.001
Age(years)				<0.001				
≤50		reference				reference		<0.001
51-65	1.986	1.770	2.229	<0.001	2.008	1.789	2.254	<0.001
>65	6.677	5.992	7.440	<0.001	6.325	5.673	7.053	<0.001
Marital status				<0.001				<0.001
Married		reference				reference		
Unmarried/NOS	1.480	1.435	1.527	<0.001	1.421	1.375	1.468	<0.001
Race				<0.001				<0.001
White		reference				reference		
Black	0.929	0.882	0.980	0.007	1.093	1.037	1.153	0.001
Other/NOS	0.711	0.664	0.761	<0.001	0.799	0.746	0.855	<0.001
Tumor location				<0.001				0.036
Right colon		reference				reference		
Left colon	0.816	0.790	0.843	<0.001	0.989	0.957	1.023	0.524
NOS	1.147	1.007	1.305	0.038	1.175	1.032	1.338	0.015
Pathological grade				<0.001				0.002
I/II		reference				reference		
III/IV	1.134	1.086	1.185	<0.001	1.076	1.029	1.125	0.001
Unknown	0.801	0.736	0.872	<0.001	0.956	0.876	1.043	0.307
Histological type				<0.001				0.099
Adenocarcinomas		reference				reference		
MCC/SRCC	1.145	1.093	1.200	<0.001	1.041	0.993	1.092	0.099
T stage				<0.001				<0.001
T1		reference				reference		
T2	1.317	1.252	1.385	<0.001	1.200	1.140	1.264	<0.001
T3	1.555	1.490	1.624	<0.001	1.481	1.415	1.549	<0.001
Chemotherapy				<0.001				<0.001
Yes		reference				reference		

No	1.617	1.527	1.712	<0.001	1.384	1.305	1.468	<0.001
**T4N0**								
**Year of diagnosis**				**0.503**				
**1999-2000**		**reference**				**NA**		
**2010-2011**	**0.967**	**0.876**	**1.067**	**0.503**				
Gender				0.954				
Female		reference				NA		
Male	1.003	0.917	1.096	0.954				
Age(years)				<0.001				<0.001
≤50		reference				reference		
51-65	1.783	1.395	2.280	<0.001	1.761	1.376	2.253	<0.001
>65	3.838	3.060	4.814	<0.001	3.364	2.670	4.238	<0.001
Marital status				<0.001				<0.001
Married		reference				reference		
Unmarried/NOS	1.416	1.294	1.548	<0.001	1.253	1.144	1.373	<0.001
Race				<0.001				0.002
White		reference				reference		
Black	1.016	0.878	1.175	0.832	1.172	1.011	1.358	0.035
Other/NOS	0.657	0.543	0.794	<0.001	0.781	0.645	0.945	0.011
Tumor location				0.776				
Right colon		reference				NA		
Left colon	0.971	0.884	1.065	0.530				
NOS	0.943	0.713	1.248	0.682				
Pathological grade				0.041				0.023
I/II		reference				reference		
III/IV	1.128	1.017	1.251	0.023	1.119	1.009	1.242	0.034
Unknown	1.215	0.903	1.634	0.199	1.338	.994	1.801	0.055
Histological type				0.372				
Adenocarcinomas		reference				NA		
MCC/SRCC	0.948	0.844	1.066	0.372				
Chemotherapy				<0.001				<0.001
Yes		reference				reference		
No	1.827	1.648	2.025	<0.001	1.380	1.239	1.537	<0.001
**T1-3N+**								
**Year of diagnosis**				**<0.001**				**<0.001**
**1999-2000**		**reference**				**reference**		
**2010-2011**	**0.789**	**0.754**	**0.827**	**<0.001**	**0.876**	**0.836**	**0.918**	**<0.001**
Gender				0.096				
Female		reference				NA		
Male	1.036	0.994	1.081	0.096				
Age(years)				<0.001				<0.001

≤50		reference				reference		
51-65	1.359	1.234	1.498	<0.001	1.375	1.247	1.515	<0.001
>65	3.152	2.885	3.443	<0.001	2.753	2.516	3.012	<0.001
Marital status				<0.001				<0.001
Married		reference				reference		
Unmarried/NOS	1.453	1.394	1.516	<0.001	1.294	1.240	1.350	<0.001
Race				<0.001				<0.001
White		reference				reference		
Black	1.028	0.964	1.097	0.402	1.156	1.083	1.235	<0.001
Other/NOS	0.787	0.727	0.852	<0.001	0.838	0.774	0.908	<0.001
Tumor location				<0.001				0.023
Right colon		reference				reference		
Left colon	0.758	0.725	0.792	<0.001	0.941	0.899	0.985	0.009
NOS	1.067	0.913	1.247	0.416	1.045	0.894	1.222	0.577
Pathological grade				<0.001				<0.001
I/II		reference				reference		
III/IV	1.285	1.226	1.346	<0.001	1.139	1.085	1.194	<0.001
Unknown	1.103	0.959	1.269	0.170	1.075	0.933	1.238	0.319
Histological type				<0.001				0.333
Adenocarcinomas		reference				reference		
MCC/SRCC	1.179	1.111	1.252	<0.001	1.030	0.970	1.095	0.333
T stage				<0.001				<0.001
T1		reference				reference		
T2	1.187	1.036	1.361	0.014	1.026	0.894	1.176	0.718
T3	1.802	1.602	2.028	<0.001	1.443	1.281	1.625	<0.001
N stage				<0.001				<0.001
N1		reference				reference		
N2	1.443	1.381	1.508	<0.001	1.518	1.451	1.588	<0.001
Chemotherapy				<0.001				<0.001
Yes		reference				reference		
No	2.019	1.936	2.106	<0.001	1.695	1.623	1.770	<0.001
**T4N+**								
**Year of diagnosis**				**<0.001**				**<0.001**
**1999-2000**		**reference**				**reference**		
**2010-2011**	**0.809**	**0.748**	**0.876**	**<0.001**	**0.859**	**0.792**	**0.931**	**<0.001**
Gender				<0.001				0.970
Female		reference				reference		
Male	0.855	0.792	0.923	<0.001	0.998	0.922	1.081	0.970
Age(years)				<0.001				<0.001
≤50		reference				reference		
51-65	1.327	1.135	1.551	<0.001	1.315	1.124	1.538	0.001

>65	2.595	2.253	2.989	<0.001	2.252	1.947	2.605	<0.001
Marital status				<0.001				<0.001
Married		reference				reference		
Unmarried/NOS	1.324	1.228	1.429	<0.001	1.189	1.098	1.288	<0.001
Race				<0.001				0.001
White		reference				reference		
Black	1.004	0.886	1.138	.950	1.150	1.012	1.306	0.032
Other/NOS	0.722	0.629	0.830	<0.001	0.820	0.713	0.944	0.006
Tumor location				<0.001				.017
Right colon		reference				reference		
Left colon	0.739	0.680	0.803	<0.001	0.883	0.811	0.962	0.004
NOS	1.006	0.796	1.271	0.961	0.979	0.774	1.238	0.858
Pathological grade				<0.001				<0.001
I/II		reference				reference		
III/IV	1.452	1.344	1.568	<0.001	1.314	1.215	1.422	<0.001
Unknown	1.177	0.895	1.548	0.244	1.138	0.862	1.504	0.361
Histological type				0.008				0.275
Adenocarcinomas		reference				reference		
MCC/SRCC	1.137	1.034	1.250	0.008	1.056	0.958	1.164	0.275
N stage				<0.001				<0.001
N1		reference				reference		
N2	1.531	1.419	1.652	<0.001	1.641	1.518	1.774	<0.001
Chemotherapy				<0.001				<0.001
Yes		reference				reference		
No	1.826	1.693	1.970	<0.001	1.559	1.440	1.689	<0.001

Table S2 Univariable and multivariable Cox regression model in patients without chemotherapy

Total cohort								
Characteristics	Univariable analysis				Multivariable analysis			
	HR	95% CI lower	95% CI upper	p-value	HR	95% CI lower	95% CI upper	p-value
**Year of diagnosis**				**<0.001**				**<0.001**
**1999-2000**		**reference**				**reference**		
**2010-2011**	**0.843**	**0.818**	**0.869**	**<0.001**	**0.933**	**0.905**	**0.962**	**<0.001**
Gender				0.488				
Female		reference				NA		
Male	1.009	0.983	1.036	0.488				
Age(years)				<0.001				<0.001
≤50		reference				reference		
51-65	1.796	1.622	1.988	<0.001	1.883	1.701	2.085	<0.001
>65	5.664	5.150	6.230	<0.001	5.481	4.982	6.031	<0.001
Marital status				<0.001				<0.001
Married		reference				reference		
Unmarried/NOS	1.525	1.485	1.566	<0.001	1.330	1.295	1.366	<0.001
Race				<0.001				<0.001
White		reference				reference		
Black	0.945	0.904	0.988	0.012	1.091	1.043	1.141	<0.001
Other/NOS	0.747	0.707	0.790	<0.001	0.794	0.751	0.840	<0.001
Tumor location				<0.001				0.315
Right colon		reference				reference		
Left colon	0.809	0.787	0.832	<0.001	0.996	0.968	1.025	0.810
NOS	1.158	1.043	1.285	.006	1.082	0.974	1.201	0.141
Pathological grade				<0.001				<0.001
I/II		reference				reference		
III/IV	1.390	1.345	1.437	<0.001	1.102	1.065	1.140	<0.001
Unknown	0.766	0.709	0.827	<0.001	0.976	0.902	1.055	0.537
Histological type				<0.001				0.602
Adenocarcinomas		reference				reference		
MCC/SRCC	1.196	1.150	1.243	<0.001	1.011	0.972	1.051	0.602
T stage				<0.001				<0.001
T1		reference				reference		
T2	1.338	1.275	1.405	<0.001	1.177	1.120	1.236	<0.001
T3	1.920	1.843	2.001	<0.001	1.467	1.405	1.532	<0.001
T4	3.167	3.004	3.338	<0.001	2.158	2.042	2.281	<0.001
Tx	1.843	1.212	2.804	.004	0.977	0.641	1.487	0.912
N stage				<0.001				<0.001

N0		reference				reference		
N1	1.787	1.726	1.849	<0.001	1.525	1.472	1.580	<0.001
N2	2.765	2.641	2.896	<0.001	2.379	2.268	2.496	<0.001
**T1-3N0**								
**Year of diagnosis**				**<0.001**				**<0.001**
**1999-2000**		**reference**				**reference**		
**2010-2011**	**0.813**	**0.782**	**0.845**	**<0.001**	**0.901**	**0.867**	**0.937**	**<0.001**
Gender				0.004				<0.001
Female		reference				reference		
Male	1.049	1.016	1.083	0.004	1.298	1.254	1.343	<0.001
Age(years)				<0.001				<0.001
≤50		reference				reference		
51-65	2.081	1.819	2.382	<0.001	2.125	1.857	2.432	<0.001
>65	7.147	6.293	8.118	<0.001	6.873	6.050	7.809	<0.001
Marital status				<0.001				<0.001
Married		reference				reference		
Unmarried/NOS	1.495	1.448	1.544	<0.001	1.436	1.388	1.486	<0.001
Race				<0.001				<0.001
White		reference				reference		
Black	0.908	0.859	0.960	0.001	1.066	1.008	1.127	0.025
Other/NOS	0.716	0.667	0.769	<0.001	0.802	0.747	0.862	<0.001
Tumor location				<0.001				0.038
Right colon		reference				reference		
Left colon	0.803	0.776	0.831	<0.001	0.979	0.945	1.014	0.231
NOS	1.153	1.005	1.322	0.042	1.163	1.014	1.334	0.031
Pathological grade				<0.001				<0.001
I/II		reference				reference		
III/IV	1.200	1.146	1.256	<0.001	1.094	1.044	1.146	<0.001
Unknown	0.762	0.698	0.831	<0.001	0.949	0.868	1.037	0.248
Histological type				<0.001				0.104
Adenocarcinomas		reference				reference		
MCC/SRCC	1.182	1.125	1.242	<0.001	1.042	0.992	1.096	0.104
T stage				<0.001				<0.001
T1		reference				reference		
T2	1.328	1.262	1.398	<0.001	1.194	1.133	1.257	<0.001
T3	1.731	1.657	1.809	<0.001	1.482	1.416	1.551	<0.001
**T4N0**								
**Year of diagnosis**				**0.061**				
**1999-2000**		**reference**				**NA**		
**2010-2011**	**1.115**	**0.995**	**1.249**	**0.061**				
Gender				0.884				

Female		reference				NA		
Male	0.992	0.895	1.100	0.884				
Age(years)				<0.001				<0.001
≤50		reference				reference		
51-65	2.005	1.346	2.986	0.001	2.065	1.386	3.077	<0.001
>65	4.083	2.805	5.944	<0.001	4.035	2.770	5.879	<0.001
Marital status				<0.001				<0.001
Married		reference				reference		
Unmarried/NOS	1.372	1.236	1.524	<0.001	1.289	1.160	1.432	<0.001
Race				0.002				0.041
White		reference				reference		
Black	0.956	0.805	1.136	0.609	1.112	0.934	1.325	0.233
Other/NOS	0.659	0.521	0.833	<0.001	0.775	0.612	0.982	0.035
Tumor location				0.656				
Right colon		reference				NA		
Left colon	1.031	0.925	1.150	0.575				
NOS	0.878	0.599	1.287	0.503				
Pathological grade				0.006				0.040
I/II		reference				reference		
III/IV	1.153	1.023	1.300	0.020	1.116	0.989	1.259	0.074
Unknown	1.542	1.079	2.204	0.017	1.430	1.000	2.045	0.050
Histological type				0.427				
Adenocarcinomas		reference				NA		
MCC/SRCC	0.946	0.826	1.084	0.427				
**T1-3N+**								
**Year of diagnosis**				**0.135**				
**1999-2000**		**reference**				**NA**		
**2010-2011**	**0.954**	**0.896**	**1.015**	**0.135**				
Gender				0.082				
Female		reference				NA		
Male	1.052	0.994	1.114	0.082				
Age(years)				<0.001				<0.001
≤50		reference				reference		
51-65	1.514	1.242	1.846	<0.001	1.591	1.305	1.940	<0.001
>65	3.854	3.212	4.623	<0.001	3.886	3.236	4.667	<0.001
Marital status				<0.001				<0.001
Married		reference				reference		
Unmarried/NOS	1.476	1.393	1.564	<0.001	1.337	1.262	1.418	<0.001
Race				<0.001				<0.001
White		reference				reference		

Black	0.993	0.907	1.088	0.882	1.128	1.029	1.236	0.010
Other/NOS	0.749	0.671	0.837	<0.001	0.782	0.699	0.874	<0.001
Tumor location				<0.001				0.583
Right colon		reference				reference		
Left colon	0.810	0.762	0.861	<0.001	0.969	0.910	1.032	0.329
NOS	1.047	0.850	1.290	0.667	1.028	0.835	1.267	0.794
Pathological grade				<0.001				0.004
I/II		reference				reference		
III/IV	1.210	1.135	1.290	<0.001	1.116	1.045	1.191	0.001
Unknown	0.903	0.740	1.101	0.314	0.962	0.788	1.175	0.707
Histological type				0.057				
Adenocarcinomas		reference				NA		
MCC/SRCC	1.082	0.998	1.174	0.057				
T stage				<0.001				<0.001
T1		reference				reference		
T2	1.124	0.936	1.351	0.211	1.017	0.846	1.223	0.857
T3	1.617	1.377	1.898	<0.001	1.378	1.173	1.619	<0.001
N stage				<0.001				<0.001
N1		reference				reference		
N2	1.401	1.316	1.491	<0.001	1.435	1.347	1.528	<0.001
**T4N+**								
**Year of diagnosis**				**0.881**				
**1999-2000**		**reference**				**NA**		
**2010-2011**	**0.992**	**0.888**	**1.108**	**0.881**				
Gender				0.021				0.730
Female		reference				reference		
Male	0.879	0.788	0.981	0.021	1.021	0.909	1.147	0.730
Age(years)				<0.001				<0.001
≤50		reference				reference		
51-65	1.284	0.923	1.784	0.138	1.261	0.906	1.754	0.169
>65	2.725	2.021	3.674	<0.001	2.676	1.977	3.621	<0.001
Marital status				<0.001				<0.001
Married		reference				reference		
Unmarried/NOS	1.355	1.214	1.513	<0.001	1.269	1.129	1.426	<0.001
Race				0.003				0.020
White		reference				reference		
Black	0.964	0.807	1.151	0.685	1.129	0.942	1.352	0.189
Other/NOS	0.697	0.568	0.856	0.001	0.781	0.634	0.962	0.020
Tumor location				<0.001				0.448
Right colon		reference				reference		
Left colon	0.779	0.690	0.879	<0.001	0.923	0.815	1.045	0.206

NOS	0.891	0.636	1.249	0.504	0.971	0.692	1.361	0.862
Pathological grade				<0.001				0.011
I/II		reference				reference		
III/IV	1.284	1.152	1.432	<0.001	1.183	1.060	1.321	0.003
Unknown	0.955	0.625	1.459	0.831	1.076	0.703	1.647	0.738
Histological type				0.966				
Adenocarcinomas		reference				NA		
MCC/SRCC	0.997	0.867	1.146	0.966				
N stage				<0.001				<0.001
N1		reference				reference		
N2	1.593	1.430	1.774	<0.001	1.639	1.471	1.827	<0.001

Table S3 The Characteristics of all colon cancer patients in 1999-2000 and 2010-2011 after PSM

**Total cohort**					
Characteristics	1999-2000(n=21730)		2010-2011(n=21730)		p
	N	%	N	%	
Gender					0.571
Female	11291	51.96%	11232	51.69%	
Male	10439	48.04%	10498	48.31%	
Age(years)					0.586
≤50	1496	6.88%	1487	6.84%	
51-65	4956	22.81%	4905	22.57%	
>65	15278	70.31%	15338	70.58%	
Marital status					0.954
Married	11626	53.50%	11632	53.53%	
Unmarried/NOS	10104	46.50%	10098	46.47%	
Race					0.291
White	18366	84.52%	18304	84.23%	
Black	1895	8.72%	1896	8.73%	
Other/NOS	1469	6.76%	1530	7.04%	
Tumor location					0.813
Right colon	13456	61.92%	13491	62.08%	
Left colon	8069	37.13%	8024	36.93%	
NOS	205	0.94%	215	0.99%	
Pathological grade					0.657
I/II	17225	79.27%	17183	79.08%	
III/IV	3985	18.34%	4025	18.52%	
Unknown	520	2.39%	522	2.40%	
Histological type					0.633
Adenocarcinomas	19344	89.02%	19375	89.16%	
MCC/SRCC	2386	10.98%	2355	10.84%	
T stage					0.689
T1	2686	12.36%	2693	12.39%	
T2	3573	16.44%	3649	16.79%	
T3	13093	60.25%	12857	59.17%	
T4	2374	10.92%	2524	11.62%	
Tx	4	0.02%	7	0.03%	
N stage					0.841
N0	14614	67.25%	14632	67.34%	
N1	4877	22.44%	4869	22.41%	
N2	2239	10.30%	2229	10.26%	
Chemotherapy					0.498
Yes	5342	24.58%	5403	24.86%	
No	16388	75.42%	16327	75.14%	

**RNE**	**10 (6-16)**		**17 (13-23)**		**<0.001**
**T1-3N0**					
	1999-2000(n=13284)		2010-2011(n=13284)		
Gender					0.314
Female	6811	51.27%	6729	50.65%	
Male	6473	48.73%	6555	49.35%	
Age(years)					0.311
≤50	799	6.01%	764	5.75%	
51-65	2799	21.07%	2773	20.87%	
>65	9686	72.91%	9747	73.37%	
Marital status					0.247
Married	7125	53.64%	7219	54.34%	
Unmarried/NOS	6159	46.36%	6065	45.66%	
Race					0.258
White	11345	85.40%	11280	84.91%	
Black	1144	8.61%	1175	8.85%	
Other/NOS	795	5.98%	829	6.24%	
Tumor location					0.981
Right colon	8212	61.82%	8214	61.83%	
Left colon	4957	37.32%	4955	37.30%	
NOS	115	0.87%	115	0.87%	
Pathological grade					0.958
I/II	11125	83.75%	11123	83.73%	
III/IV	1733	13.05%	1733	13.05%	
Unknown	426	3.21%	428	3.22%	
Histological type					0.919
Adenocarcinomas	11933	89.83%	11938	89.87%	
MCC/SRCC	1351	10.17%	1346	10.13%	
T stage					0.196
T1	2495	18.78%	2578	19.41%	
T2	3050	22.96%	3050	22.96%	
T3	7739	58.26%	7656	57.63%	
Chemotherapy					0.964
Yes	1094	8.24%	1092	8.22%	
No	12190	91.76%	12192	91.78%	
**RNE**	**10 (6-15)**		**16 (12-22)**		**<0.001**
**T4N0**					
	1999-2000(n=1157)		2010-2011(n=1157)		
Gender					1.000
Female	664	57.39%	664	57.39%	
Male	493	42.61%	493	42.61%	
Age(years)					1.000
≤50	71	6.14%	71	6.14%	

51-65	240	20.74%	240	20.74%	
>65	846	73.12%	846	73.12%	
Marital status					1.000
Married	556	48.06%	556	48.06%	
Unmarried/NOS	601	51.94%	601	51.94%	
Race					1.000
White	1015	87.73%	1015	87.73%	
Black	81	7.00%	81	7.00%	
Other/NOS	61	5.27%	61	5.27%	
Tumor location					1.000
Right colon	739	63.87%	739	63.87%	
Left colon	404	34.92%	404	34.92%	
NOS	14	1.21%	14	1.21%	
Pathological grade					1.000
I/II	897	77.53%	897	77.53%	
III/IV	255	22.04%	255	22.04%	
Unknown	5	0.43%	5	0.43%	
Histological type					1.000
Adenocarcinomas	988	85.39%	988	85.39%	
MCC/SRCC	169	14.61%	169	14.61%	
Chemotherapy					1.000
Yes	315	27.23%	315	27.23%	
No	842	72.77%	842	72.77%	
**RNE**	**10 (6-16)**		**17 (13-22)**		**<0.001**
**T1-3N+**					
	1999-2000(n=5685)		2010-2011(n=5685)		
Gender					0.985
Female	2931	51.56%	2932	51.57%	
Male	2754	48.44%	2753	48.43%	
Age(years)					0.977
≤50	468	8.23%	467	8.21%	
51-65	1456	25.61%	1456	25.61%	
>65	3761	66.16%	3762	66.17%	
Marital status					0.851
Married	3101	54.55%	3091	54.37%	
Unmarried/NOS	2584	45.45%	2594	45.63%	
Race					0.974
White	4724	83.10%	4724	83.10%	
Black	534	9.39%	536	9.43%	
Other/NOS	427	7.51%	425	7.48%	
Tumor location					0.985
Right colon	3493	61.44%	3349	58.91%	
Left colon	2150	37.82%	2139	37.63%	

NOS	42	0.74%	47	0.83%	
Pathological grade					0.920
I/II	4222	74.27%	4226	74.34%	
III/IV	1397	24.57%	1394	24.52%	
Unknown	66	1.16%	65	1.14%	
Histological type					0.952
Adenocarcinomas	5077	89.31%	5075	89.27%	
MCC/SRCC	608	10.69%	610	10.73%	
T stage					0.982
T1	171	3.01%	171	3.01%	
T2	461	8.11%	460	8.09%	
T3	5053	88.88%	5054	88.90%	
N stage					0.967
N1	4031	70.91%	4029	70.87%	
N2	1654	29.09%	1656	29.13%	
Chemotherapy					0.985
Yes	3126	54.99%	3125	54.97%	
No	2559	45.01%	2560	45.03%	
**RNE**	**11 (7-17)**		**17 (13-23)**		**<0.001**
**T4N+**					
	1999-2000(n=1177)		2010-2011(n=1177)		
Gender					1.000
Female	694	58.96%	694	58.96%	
Male	483	41.04%	483	41.04%	
Age(years)					1.000
≤50	88	7.48%	88	7.48%	
51-65	282	23.96%	282	23.96%	
>65	807	68.56%	807	68.56%	
Marital status					1.000
Married	579	49.19%	579	49.19%	
Unmarried/NOS	598	50.81%	598	50.81%	
Race					1.000
White	1026	87.17%	1026	87.17%	
Black	75	6.37%	75	6.37%	
Other/NOS	76	6.46%	76	6.46%	
Tumor location					1.000
Right colon	813	69.07%	813	69.07%	
Left colon	351	29.82%	351	29.82%	
NOS	13	1.10%	13	1.10%	
Pathological grade					1.000
I/II	723	61.43%	723	61.43%	
III/IV	449	38.15%	449	38.15%	
Unknown	5	0.42%	5	0.42%	

Histological type					1.000
Adenocarcinomas	1003	85.22%	1003	85.22%	
MCC/SRCC	174	14.78%	174	14.78%	
N stage					1.000
N1	690	58.62%	690	58.62%	
N2	487	41.38%	487	41.38%	
Chemotherapy					1.000
Yes	623	52.93%	623	52.93%	
No	554	47.07%	554	47.07%	
**RNE**	**12 (7-17)**		**18 (14-24)**		**<0.001**

Table S4 The Characteristics of colon cancer patients without chemotherapy in 1999-2000 and 2010-2011 after PSM

**Total cohort**					
Characteristics	1999-2000(n=16207)		2010-2011(n=16207)		p
	N	%	N	%	
Gender					0.982
Female	8548	52.74%	8550	52.75%	
Male	7659	47.26%	7657	47.25%	
Age(years)					0.716
≤50	721	4.45%	719	4.44%	
51-65	2971	18.33%	2940	18.14%	
>65	12515	77.22%	12548	77.42%	
Marital status					1.000
Married	8274	51.05%	8274	51.05%	
Unmarried/NOS	7933	48.95%	7933	48.95%	
Race					0.693
White	13842	85.41%	13860	85.52%	
Black	1370	8.45%	1372	8.47%	
Other/NOS	995	6.14%	975	6.02%	
Tumor location					0.860
Right colon	10305	63.58%	10295	63.52%	
Left colon	5757	35.52%	5761	35.55%	
NOS	145	0.89%	151	0.93%	
Pathological grade					0.991
I/II	13145	81.11%	13149	81.13%	
III/IV	2596	16.02%	2587	15.96%	
Unknown	466	2.88%	471	2.91%	
Histological type					0.483
Adenocarcinomas	14455	89.19%	14494	89.43%	
MCC/SRCC	1752	10.81%	1713	10.57%	
T stage					0.646
T1	2544	15.70%	2572	15.87%	
T2	3247	20.03%	3257	20.10%	
T3	8995	55.50%	8962	55.30%	
T4	1421	8.77%	1416	8.74%	
Tx	0	0.00%	0	0.00%	
N stage					0.646
N0	13066	80.62%	13028	80.39%	
N1	2267	13.99%	2298	14.18%	
N2	874	5.39%	881	5.44%	
**RNE**	**10 (6-16)**		**16 (12-22)**		**<0.001**
**T1-3N0**					
	1999-2000(n=12182)		2010-2011(n=12182)		

Gender					0.969
Female	6274	51.50%	6271	51.48%	
Male	5908	48.50%	5911	48.52%	
Age(years)					0.972
≤50	596	4.89%	597	4.90%	
51-65	2363	19.40%	2364	19.41%	
>65	9223	75.71%	9221	75.69%	
Marital status					1.000
Married	6453	52.97%	6453	52.97%	
Unmarried/NOS	5729	47.03%	5729	47.03%	
Race					0.942
White	10420	85.54%	10423	85.56%	
Black	1032	8.47%	1032	8.47%	
Other/NOS	730	5.99%	727	5.97%	
Tumor location					0.970
Right colon	7621	62.56%	7623	62.58%	
Left colon	4450	36.53%	4449	36.52%	
NOS	111	0.91%	110	0.90%	
Pathological grade					0.936
I/II	10234	84.01%	10231	83.98%	
III/IV	1525	12.52%	1525	12.52%	
Unknown	423	3.47%	426	3.50%	
Histological type					1.000
Adenocarcinomas	10940	89.80%	10940	89.80%	
MCC/SRCC	1242	10.20%	1242	10.20%	
T stage					1.000
T1	2474	20.31%	2474	20.31%	
T2	3015	24.75%	3015	24.75%	
T3	6693	54.94%	6693	54.94%	
**RNE**	**10 (6-15)**		**16 (12-22)**		**<0.001**
**T4N0**					
	1999-2000(n=842)		2010-2011(n=842)		
Gender					1.000
Female	500	59.38%	500	59.38%	
Male	342	40.62%	342	40.62%	
Age(years)					0.915
≤50	21	2.49%	22	2.61%	
51-65	119	14.13%	119	14.13%	
>65	702	83.37%	701	83.25%	
Marital status					0.961
Married	358	42.52%	359	42.64%	
Unmarried/NOS	484	57.48%	483	57.36%	
Race					0.921

White	739	87.77%	738	87.65%	
Black	62	7.36%	62	7.36%	
Other/NOS	41	4.87%	42	4.99%	
Tumor location					0.961
Right colon	561	66.63%	560	66.51%	
Left colon	274	32.54%	275	32.66%	
NOS	7	0.83%	7	0.83%	
Pathological grade					1.000
I/II	652	77.43%	652	77.43%	
III/IV	186	22.09%	186	22.09%	
Unknown	4	0.48%	4	0.48%	
Histological type					0.945
Adenocarcinomas	719	85.39%	720	85.51%	
MCC/SRCC	123	14.61%	122	14.49%	
**RNE**	**10 (6-16)**		**17 (13-22)**		**<0.001**
**T1-3N+**					
	1999-2000(n=2565)		2010-2011(n=2565)		
Gender					0.933
Female	1401	54.62%	1398	54.50%	
Male	1164	45.38%	1167	45.50%	
Age(years)					0.931
≤50	85	3.31%	84	3.27%	
51-65	380	14.81%	379	14.78%	
>65	2100	81.87%	2102	81.95%	
Marital status					0.467
Married	1213	47.29%	1187	46.28%	
Unmarried/NOS	1352	52.71%	1378	53.72%	
Race					0.980
White	2160	84.21%	2159	84.17%	
Black	234	9.12%	235	9.16%	
Other/NOS	171	6.67%	171	6.67%	
Tumor location					1.000
Right colon	1671	65.15%	1671	65.15%	
Left colon	871	33.96%	871	33.96%	
NOS	23	0.90%	23	0.90%	
Pathological grade					0.338
I/II	1894	73.84%	1865	72.71%	
III/IV	636	24.80%	661	25.77%	
Unknown	35	1.36%	39	1.52%	
Histological type					1.000
Adenocarcinomas	2292	89.36%	2292	89.36%	
MCC/SRCC	273	10.64%	273	10.64%	
T stage					0.335

T1	63	2.46%	62	2.42%	
T2	231	9.01%	205	7.99%	
T3	2271	88.54%	2298	89.59%	
N stage					0.412
N1	1893	73.80%	1867	72.79%	
N2	672	26.20%	698	27.21%	
**RNE**	**11 (7-16)**		**17 (13-23)**		**<0.001**
**T4N+**					
	1999-2000(n=554)		2010-2011(n=554)		
Gender					1.000
Female	349	63.00%	349	63.00%	
Male	205	37.00%	205	37.00%	
Age(years)					1.000
≤50	7	1.26%	7	1.26%	
51-65	70	12.64%	70	12.64%	
>65	477	86.10%	477	86.10%	
Marital status					1.000
Married	202	36.46%	202	36.46%	
Unmarried/NOS	352	63.54%	352	63.54%	
Race					1.000
White	487	87.91%	487	87.91%	
Black	38	6.86%	38	6.86%	
Other/NOS	29	5.23%	29	5.23%	
Tumor location					1.000
Right colon	414	74.73%	414	74.73%	
Left colon	133	24.01%	133	24.01%	
NOS	7	1.26%	7	1.26%	
Pathological grade					1.000
I/II	344	62.09%	344	62.09%	
III/IV	209	37.73%	209	37.73%	
Unknown	1	0.18%	1	0.18%	
Histological type					1.000
Adenocarcinomas	489	88.27%	489	88.27%	
MCC/SRCC	65	11.73%	65	11.73%	
N stage					1.000
N1	345	62.27%	345	62.27%	
N2	209	37.73%	209	37.73%	
**RNE**	**11 (7-17)**		**16 (13-21)**		**<0.001**

Table S5 Univariable and multivariable Cox regression model in T1-3N0 patients with RNE ≥12

**T1-3N0 patients with RNE ≥12**								
Characteristics	Univariable analysis				Multivariable analysis			
	HR	95% CI lower	95% CI upper	p-value	HR	95% CI lower	95% CI upper	p-value
**Year of diagnosis**				**<0.001**				**0.138**
**1999-2000**		**reference**				**reference**		
**2010-2011**	**0.901**	**0.855**	**0.949**	**<0.001**	**0.961**	**0.911**	**1.013**	**0.138**
Gender				0.002				<0.001
Female		reference				reference		
Male	1.071	1.026	1.118	0.002	1.293	1.236	1.353	<0.001
Age(years)				<0.001				<0.001
≤50		reference				reference		
51-65	2.031	1.746	2.364	<0.001	2.040	1.753	2.375	<0.001
>65	7.023	6.096	8.090	<0.001	6.673	5.787	7.696	<0.001
Marital status				<0.001				<0.001
Married		reference				reference		
Unmarried/NOS	1.501	1.438	1.567	<0.001	1.444	1.380	1.511	<0.001
Race				<0.001				<0.001
White		reference				reference		
Black	0.933	0.867	1.003	0.059	1.126	1.047	1.212	0.001
Other/NOS	0.651	0.590	0.718	<0.001	0.748	0.678	0.826	<0.001
Tumor location				<0.001				0.296
Right colon		reference				reference		
Left colon	0.830	0.791	0.871	<0.001	1.013	0.964	1.064	0.617
NOS	1.089	0.918	1.292	0.326	1.141	0.962	1.353	0.131
Pathological grade				<0.001				0.084
I/II		reference				reference		
III/IV	1.132	1.068	1.199	<0.001	1.066	1.005	1.131	0.032
Unknown	0.822	0.716	0.942	0.005	0.966	0.840	1.110	0.626
Histological type				<0.001				0.191
Adenocarcinomas		reference				reference		
MCC/SRCC	1.133	1.063	1.209	<0.001	1.045	0.979	1.115	0.191
T stage				<0.001				<0.001
T1		reference				reference		
T2	1.355	1.254	1.465	<0.001	1.220	1.127	1.320	<0.001

T3	1.606	1.501	1.718	<0.001	1.515	1.413	1.624	<0.001
Chemotherapy				<0.001				<0.001
Yes		reference				reference		
No/Unknown	1.755	1.618	1.904	<0.001	1.404	1.291	1.527	<0.001
**T1-3N0 patients with RNE ≥12 who did not receive chemotherapy**								
**Year of diagnosis**				**<0.001**				**0.091**
**1999-2000**		**reference**				**reference**		
**2010-2011**	**0.862**	**0.816**	**0.910**	**<0.001**	**0.954**	**0.903**	**1.008**	**0.091**
Gender				0.016				<0.001
Female		reference				reference		
Male	1.056	1.010	1.105	0.016	1.283	1.224	1.345	<0.001
Age(years)				<0.001				<0.001
≤50		reference				reference		
51-65	2.078	1.744	2.476	<0.001	2.109	1.770	2.514	<0.001
>65	7.240	6.140	8.536	<0.001	7.002	5.935	8.260	<0.001
Marital status				<0.001				<0.001
Married		reference				reference		
Unmarried/NOS	1.515	1.449	1.585	<0.001	1.455	1.388	1.525	<0.001
Race				<0.001				<0.001
White		reference				reference		
Black	0.893	0.827	0.964	0.004	1.075	0.995	1.161	0.068
Other/NOS	0.658	0.594	0.729	<0.001	0.746	0.673	0.827	<0.001
Tumor location				<0.001				0.244
Right colon		reference				reference		
Left colon	0.819	0.779	0.862	<0.001	0.994	0.944	1.047	0.832
NOS	1.125	0.942	1.343	0.192	1.161	0.972	1.386	0.100
Pathological grade				<0.001				0.023
I/II		reference				reference		
III/IV	1.201	1.130	1.276	<0.001	1.083	1.018	1.152	0.011
Unknown	0.768	0.666	0.886	<0.001	0.937	0.811	1.083	0.379
Histological type				<0.001				0.264
Adenocarcinomas		reference				reference		
MCC/SRCC	1.150	1.075	1.230	<0.001	1.040	0.971	1.113	0.264
T stage				<0.001				<0.001
T1		reference				reference		
T2	1.363	1.261	1.475	<0.001	1.214	1.121	1.314	<0.001

T3	1.775	1.657	1.901	<0.001	1.517	1.413	1.627	<0.001

Table S6 The Characteristics of T1-3N0 colon cancer patients with RNE ≥12 in 1999-2000 and 2010-2011 after PSM

T1-3N0 patients with RNE ≥12					
Characteristics	1999-2000(n=5724)		2010-2011(n=5724)		p
	N	%	N	%	
Gender					0.955
Female	3080	53.81%	3077	53.76%	
Male	2644	46.19%	2647	46.24%	
Age(years)					0.928
≤50	438	7.65%	437	7.63%	
51-65	1237	21.61%	1233	21.54%	
>65	4049	70.74%	4054	70.82%	
Marital status					0.985
Married	3023	52.81%	3024	52.83%	
Unmarried/NOS	2701	47.19%	2700	47.17%	
Race					0.971
White	4909	85.76%	4910	85.78%	
Black	490	8.56%	490	8.56%	
Other/NOS	325	5.68%	324	5.66%	
Tumor location					0.969
Right colon	4010	70.06%	4008	70.02%	
Left colon	1650	28.83%	1652	28.86%	
NOS	64	1.12%	64	1.12%	
Pathological grade					0.921
I/II	4631	80.90%	4625	80.80%	
III/IV	945	16.51%	952	16.63%	
Unknown	148	2.59%	147	2.57%	
Histological type					0.910
Adenocarcinomas	5012	87.56%	5016	87.63%	
MCC/SRCC	712	12.44%	708	12.37%	
T stage					0.903
T1	667	11.65%	671	11.72%	
T2	1197	20.91%	1197	20.91%	
T3	3860	67.44%	3855	67.35%	
Chemotherapy					0.931
Yes	669	11.69%	672	11.74%	
No	5055	88.31%	5052	88.26%	
**RNE**	**17 (14-22)**		**19 (15-24)**		**<0.001**
**T1-3N0 patients with RNE ≥12 who did not receive chemotherapy**					
	1999-2000(n=5054)		2010-2011(n=5054)		
Gender					1.000
Female	2747	54.35%	2747	54.35%	

Male	2307	45.65%	2307	45.65%	
Age(years)					0.986
≤50	313	6.19%	313	6.19%	
51-65	990	19.59%	991	19.61%	
>65	3751	74.22%	3750	74.20%	
Marital status					1.000
Married	2614	51.72%	2614	51.72%	
Unmarried/NOS	2440	48.28%	2440	48.28%	
Race					0.969
White	4345	85.97%	4344	85.95%	
Black	430	8.51%	430	8.51%	
Other/NOS	279	5.52%	280	5.54%	
Tumor location					0.984
Right colon	3600	71.23%	3599	71.21%	
Left colon	1392	27.54%	1393	27.56%	
NOS	62	1.23%	62	1.23%	
Pathological grade					0.967
I/II	4107	81.26%	4106	81.24%	
III/IV	802	15.87%	802	15.87%	
Unknown	145	2.87%	146	2.89%	
Histological type					1.000
Adenocarcinomas	4406	87.18%	4406	87.18%	
MCC/SRCC	648	12.82%	648	12.82%	
T stage					1.000
T1	661	13.08%	661	13.08%	
T2	1179	23.33%	1179	23.33%	
T3	3214	63.59%	3214	63.59%	
**RNE**	**17 (14-22)**		**18 (15-24)**		**<0.001**
